# Half-sandwich nickel(II) complexes bearing 1,3-di(cycloalkyl)imidazol-2-ylidene ligands

**DOI:** 10.3762/bjoc.11.235

**Published:** 2015-11-12

**Authors:** Johnathon Yau, Kaarel E Hunt, Laura McDougall, Alan R Kennedy, David J Nelson

**Affiliations:** 1 WestCHEM Department of Pure and Applied Chemistry, University of Strathclyde, Thomas Graham Building, 295 Cathedral Street, Glasgow G1 1XL, UK

**Keywords:** catalysis, cross-coupling, *N*-heterocyclic carbenes, nickel

## Abstract

Two new nickel catalysts have been prepared using a convenient procedure where nickelocene, the NHC·HBF_4_ salts, and [Et_4_N]Cl were heated in THF using microwave irradiation. The resulting [NiCl(Cp)(NHC)] complexes are air- and moisture stable in the solid state, and represent two new members of this valuable and practical class of nickel catalysts. The new species were fully characterised using methods including NMR spectroscopy and X-ray crystallography. When tested in model Suzuki–Miyaura cross-coupling reactions, these complexes were found to be active for the cross-coupling of aryl bromides and aryl chlorides.

## Introduction

**Scheme 1:**
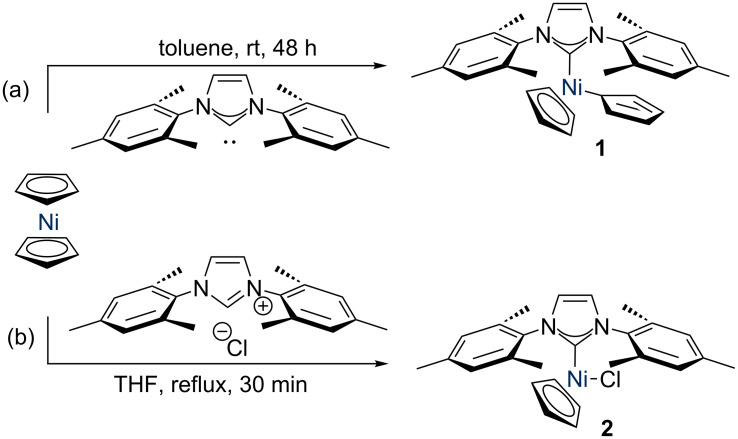
Synthesis of [Ni(η^1^-Cp)(η^5^-Cp)(IMes)] (**1**) and [NiCl(Cp)(IMes)] (**2**).

Nickel catalysis is currently an area of great interest, due to the potential for nickel to replace palladium in some catalytic processes, as well as its ability to perform a much wider range of reactions [1]. Nickel complexes bearing N-heterocyclic carbenes (NHCs) [2] are an interesting class of catalysts [3–4], due to the fascinating characteristics of NHC ligands, which can be designed to have a wide range of steric and electronic properties [5–7]. Nickel catalysts of the form [Ni(Cp)X(NHC)] have been shown to be versatile and relatively easy-to-handle nickel pre-catalysts for a range of transformations; these species are typically stable to air and moisture in the solid state and are therefore practical and accessible catalysts for a range of researchers [8]. Initial complexes of this motif were disclosed by Cowley and Jones, who prepared [Ni(η^1^-Cp)(η^5^-Cp)(IMes)] (**1**) from the reaction of the free carbene with nickelocene (IMes = 1,3-bis(2,4,6-trimethylphenyl)imidazol-2-ylidene) (Scheme 1a) [9]. Complexes of the form [NiCl(Cp)(NHC)], such as complex **2**, are typically prepared by simply heating nickelocene with the corresponding NHC·HCl salt, rendering these species highly accessible (Scheme 1b) [10]. After the initial work by Cowley and Jones, various other researchers have disclosed complexes of this form and tested them in cross-coupling reactions such as Buchwald–Hartwig amination [11], Suzuki–Miyaura cross-coupling [12], and ketone α-arylation [3,13]. These species can also catalyse hydrosilylation reactions [14].

Chatani and co-workers have recently reported that ICy (ICy = 1,3-dicyclohexylimidazol-2-ylidene) is a superior ligand for the cross-coupling of aryl and benzyl methyl ethers with arylboronic acid esters, when used as the HCl or HBF_4_ salt combined with [Ni(COD)_2_] [15–17]. In addition, a [Ni(OAc)_2_]/ICy·HCl system was found to allow the cross-coupling of Grignard reagents with aryl ethers [18].

However, the identity of the active catalyst, which is formed in situ in these reactions, is as yet unknown, and might be a mono- or bis-NHC complex. We therefore decided to prepare and test [NiCl(Cp)(ICy)] (**3**) (closely related to [NiCl(Cp)(IDD)] (**4**)) in some model catalytic reactions, to discover whether the favourable properties of ICy in cross-coupling catalysis could be combined with the ease of synthesis and handling of the nickel half-sandwich motif. Advantages to the use of well-defined catalytic species include that the catalyst and ligand are delivered in a specific and known ratio (in this case 1:1), and that there is no need for a ‘pre-reaction’ to combine ligand and metal which are both typically added in relatively low concentrations.

## Results and Discussion

### Catalyst synthesis

**Scheme 2:**
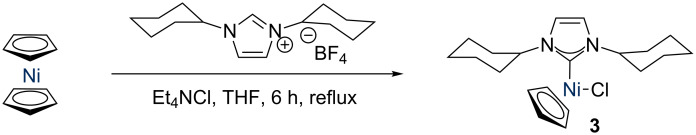
Synthesis of [NiCl(Cp)(ICy)] using conventional heating.

Nolan reported that the ICy and I*t*-Bu complexes could not be prepared by heating nickelocene with NHC·HCl salts (I*t*-Bu = 1,3-di-*tert*-butylimidazol-2-ylidene) [19]. In addition, these salts are highly hygroscopic, and therefore difficult to prepare and purify. The tetrafluoroborate salts can be prepared in a one-pot procedure and are easy-to-handle non-hygroscopic white powders, so our first aim was to synthesise the target complexes from NHC·HBF_4_ and nickelocene. We were pleased to find that we could prepare [NiCl(Cp)(ICy)] (**3**) by adding [Et_4_N]Cl to a suspension of ICy·HBF_4_ and [Ni(Cp)_2_] in THF and heating the suspension at reflux for 6 h under an argon atmosphere (Scheme 2), in a manner analogous to that recently reported by Albrecht for the synthesis of triazolylidene-based complexes [20]. However, the yield was rather poor (ca. 20%), so a better route was desired. Changing the solvent to refluxing anhydrous 1,4-dioxane did not improve yields, nor did increased reaction times, or the use of a slight excess of nickelocene. We suspected that the product might be thermally unstable in solution, and that competing decomposition might be reducing the yield. To test this, a purified sample of **3** was subjected to [Et_4_N]Cl in refluxing anhydrous 1,4-dioxane for 6 h; the deep red solution turned pale and yielded a black precipitate, confirming this hypothesis.

**Scheme 3:**
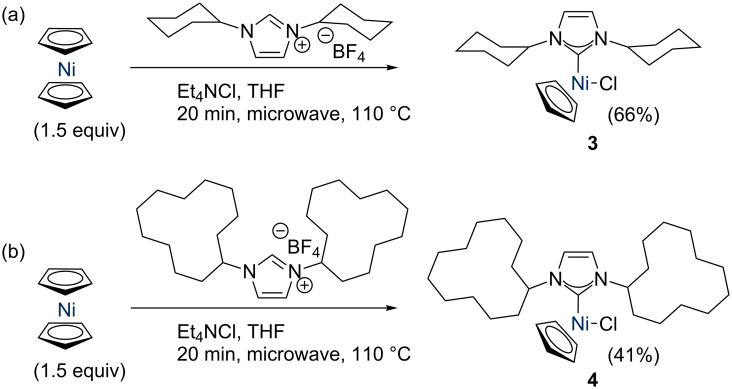
Synthesis of (a) [NiCl(Cp)(ICy)] (**3**) and (b) [NiCl(Cp)(IDD)] (**4**) with microwave heating.

Navarro reported that [NiCl(Cp)(NHC)] complexes can be prepared in much shorter reaction times by using microwave heating [21]. As the microwave apparatus heats the solvent directly, rather than applying heat to the outer walls of a glass vessel, it was proposed that this might allow for better yields. Optimisation of the reaction conditions (20 min at 110 °C in THF with 1.5 equiv nickelocene) allowed complex **3** to be isolated after work-up in analytically pure form, in 66% yield (Scheme 3a). Slightly lower yields were obtained when less nickelocene was added (43% yield, 1.1 equiv), while a further increase in stoichiometry to 2 equiv did not improve the yield.

With this new complex in hand, structurally similar examples were approached using the same methodology. IDD is a larger analogue of ICy (IDD = 1,3-dicyclododecylimidazol-2-ylidene) [22]; while it typically presents a similar steric profile to ICy in calculations of percent buried volume (% V_bur_) using solid state structures [6], it is much larger and more flexible. I*t*-Bu possesses significant steric bulk close to the metal centre, and has been known to allow the isolation of the interesting 16 electron three-coordinated [Ni(CO)_2_(I*t*-Bu)] complex (I*t*-Bu = 1,3-di-*tert-*butylimidazol-2-ylidene) [23]. It has also been reported to trigger spontaneous C–H activation upon coordination to Rh(I) and Ir(I) complexes, leading to 14 electron Rh(III) and Ir(III) species [24–25]. While the methodology applied to ICy worked for IDD (Scheme 3b), repeated attempts to isolate the I*t*-Bu analogue were unsuccessful. Similarly, attempts to first prepare [Ni(η^1^-Cp)(η^5^-Cp)(I*t*-Bu)] by the reaction of free I*t*-Bu with [NiCp_2_] (analogous to Cowley’s method) [9], envisaging subsequent replacement of the Cp ligand with chloride, were not successful.

**Figure 1:**
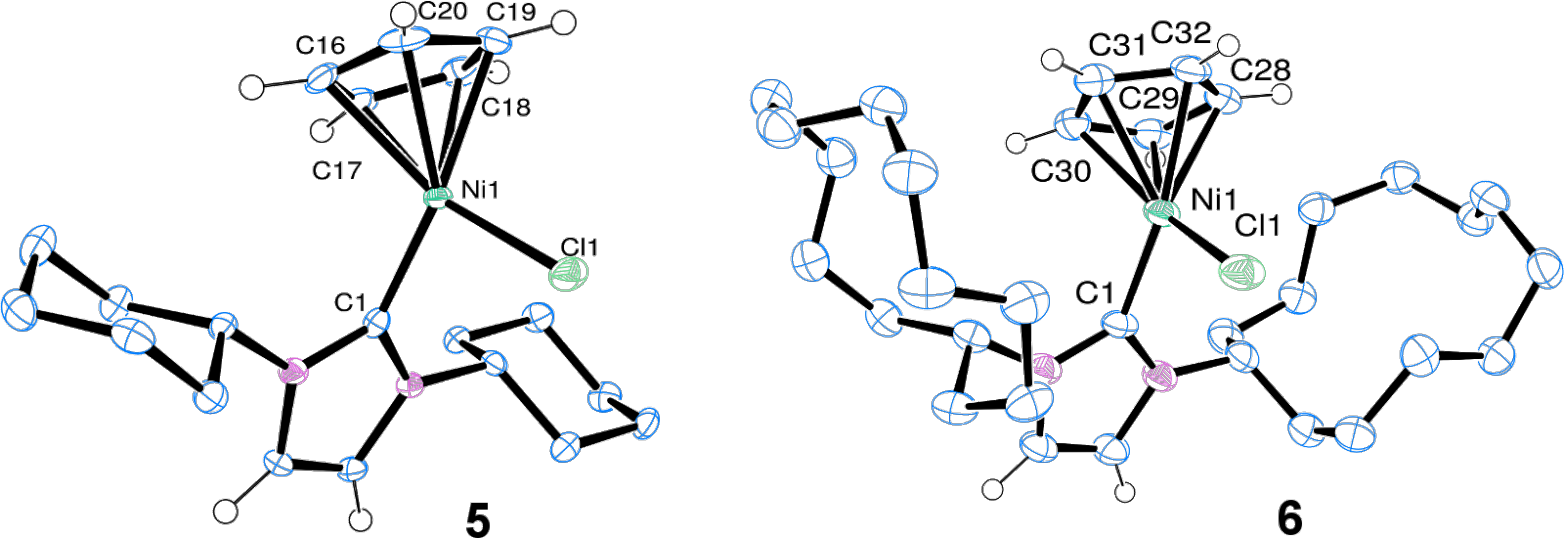
Molecular structures of complexes [NiCl(Cp)(ICy)] (**3**) (left) and [NiCl(Cp)(IDD)] (**4**) (right) as determined by single crystal X-ray diffraction. Displacement ellipsoids are drawn at 50% probability. Most H atoms are excluded for clarity.

The two new complexes were characterised by ^1^H and ^13^C{^1^H} NMR spectroscopy, elemental analysis and X-ray crystallography (Figure 1). Selected crystallographic data can be found in Table 1, and some key bond lengths in Table 2. X-ray quality crystals were obtained by slow diffusion of pentane into a DCM solution of each complex.

**Table 1:** Experimental data for single-crystal X-ray diffraction analyses of [NiCl(Cp)(ICy)] and [NiCl(Cp)(IDD)].

Structure	[NiCl(Cp)(ICy)] (**3**)	[NiCl(Cp)(IDD)] (**4**)

CCDC ref.	1049879	1049880
Formula	C_20_H_29_ClN_2_Ni	C_32_H_53_ClN_2_Ni
Formula wt	391.61 g mol^−1^	559.92 g mol^−1^
Crystal system	orthorhombic	monoclinic
*a*	16.3306(3) Å	8.5685(2) Å
*b*	10.2549(2) Å	13.9639(3) Å
*c*	11.1879(2) Å	25.1631(6) Å
β	90°	97.719(2)°
V	1873.62(6) Å^3^	2983.48(12) Å^3^
Space group	*Pca*2_1_	*P*2_1_/*n*
Z	4	4
μ	1.182 mm^−1^	1.912 mm^−1^
Reflns collected	8979	11638
Reflns unique	4499	5828
Reflns observed	4029	4521
R_int_	0.0316	0.0313
Goodness of fit	1.021	1.036
R1 (*I* > 2σ(I))	0.0336	0.0427
wR2	0.0683	0.1145

**Table 2:** Selected bond distances (units Å).

[NiCl(Cp)(ICy)] (**3**)		[NiCl(Cp)(IDD)] (**4**)	

Ni(1)–Cl(1)	2.1884(7)	Ni(1)–Cl(1)	2.1833(7)
Ni(1)–C(16)	2.136(3)	Ni(1)–C(28)	2.181(2)
Ni(1)–C(17)	2.056(3)	Ni(1)–C(29)	2.095(2)
Ni(1)–C(18)	2.160(3)	Ni(1)–C(30)	2.091(2)
Ni(1)–C(19)	2.137(3)	Ni(1)–C(31)	2.181(2)
Ni(1)–C(20)	2.192(3)	Ni(1)–C(32)	2.142(2)

The ^1^H NMR spectra show the expected features; a sharp singlet for the cyclopentadienyl ligand in each complex suggests that this ligand rotates faster than the NMR timescale, while a sharp singlet was also observed for the imidazol-2- ylidene backbone protons. The cycloalkyl nature of the N-substituents results in most of the proton signals for these species appearing as broad multiplets, even at high fields. The methine signal for the cycloalkyl substituents is discrete, appearing at δ_H_ = 6.01 ppm for ICy and δ_H_ = 6.28 ppm for IDD as a triplet of triplets and an apparent quintet, respectively. In the carbon NMR spectra, the imidazol-2-ylidene C2 signals resonate at δ_C_ = 157.0 ppm (ICy) or 158.0 ppm (IDD), compared to ca. 200 ppm for complexes of saturated *N*,*N*-diarylimidazol-2-ylidenes and ca. 170 ppm for their unsaturated counterparts [19]. This difference in δ_C_ might result from the larger net electron-donating ability of ICy and IDD, as inferred from their lower TEP compared to IPr, IMes, and IPr*, for example [7,26–27].

The crystal structure data for [NiCl(Cp)(ICy)] (**3**) reveal five different Ni–C distances between the nickel centre and the cyclopentadienyl ligand, spanning a range of ca. 0.14 Å. These bond lengths are indicative of distortion from ideal η^5^-geometry towards η^1^,η^4^-geometry (Table 2) [28]. In [NiCl(Cp)(IDD)] (**4**), the distances span a smaller range (ca. 0.09 Å). In both cases, the Cl–Ni–C1 angle is ca. 93–94°. The cyclohexyl rings adopt a chair conformation, while the cyclododecyl rings droop down around the metal centre, with the shortest Cl–H distance being ca. 2.9 Å, approximately the sum of van der Waals radii of the two atoms.

### Assessment of catalytic activity

**Scheme 4:**
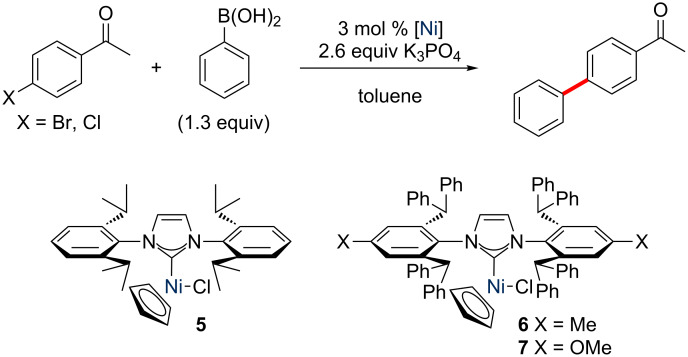
Model Suzuki–Miyaura reaction for the evaluation of the new complexes as cross-coupling pre-catalysts.

With these new complexes in hand, their activity in some model cross-coupling reactions was examined. Ritleng and Chetcuti have deployed [Ni(Cp)(X)(NHC)] complexes in Suzuki–Miyaura cross-coupling reactions of 4’-bromoacetophenone and 4’-chloroacetophenone with phenylboronic acid (Scheme 4) [12]. It was therefore decided to study these reactions as part of our preliminary evaluation of these new complexes as potential pre-catalysts for cross-coupling reactions. The reactions were conducted in duplicate, and were analysed by ^1^H NMR methods to calculate conversion. The new ICy- and IDD-bearing complexes were benchmarked against [NiCl(Cp)(IPr)] (**5**) due to the ubiquity of this carbene in transition metal-mediated catalysis [29–30], and [NiCl(Cp)(IPr*)] (**6**) and [NiCl(Cp)(IPr*^OMe^)] (**7**) due to their demonstrated competence as catalysts for the arylation of anilines [11].

The results from the cross-coupling reactions with 4’-bromo- and 4’-chloroacetophenone are summarised in Table 3; running the latter reaction for more than 2 h did not lead to significant increases in conversion.

**Table 3:** Results of test reactions with 4’-bromo- and 4’-chloroacetophenone, conducted using the conditions detailed in Scheme 4.^a^

Complex	X = Br		X = Cl		
		
	90 °C, 0.5 h		110 °C, 0.5 h	110 °C, 2 h	110 °C, 4 h

[NiCl(Cp)(IPr)] (**5**)	41%		39%	39%	39%
[NiCl(Cp)(ICy)] (**3**)	69%		38%	49%	65%
[NiCl(Cp)(IDD)] (**4**)	40%		43%	53%	54%
[NiCl(Cp)(IPr*)] (**6**)	22%		20%	27%	ND^b^
[NiCl(Cp)(IPr*^OMe^) (**7**)	32%		ND^b^	ND^b^	ND^b^

^a^All reactions conducted with 3 mol % of pre-catalyst. The conversion to the cross-coupling product was determined in each case by integration of the ^1^H NMR spectrum of the reaction mixture. Quoted results are an average of at least two independent experiments. ^b^Not determined.

Unfortunately, we were unable to reproduce the literature conversion with [NiCl(Cp)(IPr)] (87%) despite multiple attempts by different chemists. Different batches of toluene and base (with different water content) were screened, but we consistently achieved much lower conversions. Nevertheless, this provided a platform from which we could assess the new catalysts.

Surprisingly, the IPr* and IPr*^OMe^-bearing complexes performed rather poorly under these conditions. It was noted that in the Suzuki–Miyaura reactions, the catalysts bearing ICy or IDD changed colour (pink to orange-brown) more quickly, which may be suggestive of faster initiation.

Intrigued as to whether the more electron-donating nature of ICy and IDD, as inferred from their lower TEP [22,31], might influence their oxidative addition reactivity, the cross-coupling of 4’-chloroacetophenone and phenylboronic acid was investigated. These experiments suggest that the ICy and IDD complexes are indeed slightly better catalysts for the cross-coupling of more challenging electrophiles. However, extension of these studies to 4-chloroanisole which is a challenging and electron-rich aryl chloride substrate, led to disappointing conversions (ca*.* 15% with [NiCl(Cp)(ICy)] (**3**)). This may be due to the thermal sensitivity of these complexes, or may suggest that the active species in Chatani’s work is in fact a bis(NHC) complex. Further work is underway in our laboratory to understand the effect of NHC structure on cross-coupling reactivity, and to prepare and evaluate bis(ICy) complexes in catalysis.

## Conclusion

We have prepared two new half-sandwich Ni(II) complexes, which until recently had been missing relatives in this practical and useful family of pre-catalysts. X-ray crystallographic analyses suggest a tendency towards η^1^,η^4^-coordination of the cyclopentadienyl ligand.

Preliminary catalyst testing in model Suzuki–Miyaura reactions suggest that these more electron-donating ligands yield complexes that are slightly more active for the cross-coupling of aryl chlorides but less thermally stable. Further studies to fully explore and apply the reactivity of the new complexes are presently underway in our laboratory.

## Experimental

**General.** ICy·HBF_4_ and I*t*-Bu·HBF_4_ were prepared according to literature procedures [22,25]. Complexes [NiCl(Cp)(IPr)], [NiCl(Cp)(IPr*)] and [NiCl(Cp)(IPr*^OMe^)] were prepared according to literature procedures [11,19]. Nickelocene was purchased from Strem or Alfa Aesar and stored at −40 °C in the glovebox freezer. Anhydrous, oxygen-free THF and toluene were obtained from an Innovative Technologies PureSolv system (<10 ppm H_2_O, as measured by regular Karl Fischer analyses). Anhydrous 1,4-dioxane was obtained from Sigma-Aldrich and sparged with argon before use. [Et_4_N]Cl was purchased from Alfa Aesar and dried by heating under vacuum. Reactions under microwave irradiation were carried out using a Biotage apparatus in crimp-cap microwave vials equipped with magnetic stirrer bars.

NMR spectra were acquired using Bruker AV3-400, AV-400, AV3-500HD and AVII-600 spectrometers at 300 K. ^1^H NMR chemical shifts are reported in ppm referenced to residual solvent signals, while ^13^C{^1^H} NMR chemical shifts are reported referenced to deuterated solvent signals [32]. 2D experiments such as [^1^H,^1^H] COSY, [^1^H,^13^C] HSQC and [^1^H,^13^C] HMBC were used where necessary to assign chemical shifts. Elemental analyses were conducted using a Perkin Elmer 2400 Series II instrument. X-ray crystallographic analyses were undertaken with samples mounted in oil at 123(2) K using an Oxford Diffraction diffractometer equipped with a CCD detector. All structures were refined against F^2^ and to convergence using all unique reflections and the program Shelxl-97 [33].

**IDD·HBF_4_**. Paraformaldehyde (411 mg, 13.7 mmol) was suspended in toluene (25 mL) and cyclododecylamine (2.51 g, 13.7 mmol) was added. The reaction was stirred at room temperature for 3 h, and then cooled to 0 °C in an ice bath. A further portion of cyclododecylamine (2.48 g, 13.5 mmol) was added, followed by the careful addition of aqueous HBF_4_ solution (48 wt %, 2 mL, 1.34 g, 15.3 mmol). After the reaction was warmed to room temperature, aqueous glyoxal solution (40 wt %, 2 mL, 1.01 g, 17.4 mmol) was added and the reaction was heated to 40 °C and stirred vigorously overnight. The reaction was quenched with sat. aqueous NaHCO_3_ solution and filtered on a sintered frit. The resulting solid was washed on the frit with diethyl ether until the solid was white. Drying in a vacuum oven overnight at 50 °C yielded the title compound as a free-flowing white solid. Yield: 6.32 g (12.9 mmol, 95%). ^1^H NMR (CDCl_3_, 400 MHz) δ_H_ 9.13 (s, 1H, NCHN), 7.30 (s, 2H, N(CH)_2_N), 4.58 (quint., ^3^*J*_HH_ = 5.6 Hz, 2H, NCHR_2_), 2.16–2.00 (m, 4H, CH_2_), 1.82–1.71 (m, 4H, CH_2_), 1.56–1.24 (m, 36H, CH_2_); ^13^C{^1^H} NMR (CDCl_3_, 151 MHz) δ_C_ 134.5 (NCN), 120.9 (N(CH)_2_N), 59.0 (NCHR_2_), 30.4 (CH_2_), 23.6 (CH_2_), 23.5 (CH_2_), 23.4 (CH_2_), 21.5 (CH_2_). Even at high field (14.1 T), not all CH_2_ signals could be successfully resolved. Anal. calcd. for C_27_H_49_BF_4_N_2_: C, 66.39; H, 10.11; N, 5.73; found: C, 65.96; H, 10.47; N, 5.76;

**[NiCl(Cp)(ICy)].** In the glovebox, a microwave vial with stir bar was charged with nickelocene (75.1 mg, 0.398 mmol, 1.5 equiv), ICy·HBF_4_ (84.6 mg, 0.263 mmol, 1 equiv) and [Et_4_N]Cl (43.7 mg, 0.264 mmol, 1 equiv) and the cap was secured with parafilm. Outside the glovebox, under a flow of argon, anhydrous THF (3 mL) was added and the vial was sealed with a crimp cap. The reaction was heated to 110 °C for 20 min in the microwave, during which time the solution changed the colour from green to red/purple. The THF was removed and the residue was taken up in hot toluene and filtered. The volume was reduced to ca. 1 mL, and then hexane was added to precipitate the product. The green solution was decanted, and the solid was washed with hexane and dried under high vacuum to yield the product as a pink powder. Yield: 68.6 mg (0.175 mmol, 66%). ^1^H NMR (CDCl_3_, 400 MHz) δ_H_ 6.94 (s, 2H, N(CH)_2_N), 6.01 (tt, ^3^*J*_HH_ = 12, 3.9 Hz, 2H, NCHR_2_), 5.20 (s, 5H, CpH), 2.49–2.36 (m, 2H, Cy CH_2_), 2.10–1.92 (m, 6H, Cy CH_2_), 1.92–1.83 (m, 2H, Cy CH_2_), 1.81–1.47 (m, 8H, Cy CH_2_), 1.38–1.22 (2H, m, Cy CH_2_); ^13^C{^1^H} NMR (CDCl_3_, 101 MHz) δ_C_ 157.0 (NCN), 118.9 (N(CH)_2_N), 91.6 (Cp CH), 61.1 (NCHR_2_), 34.4 (Cy CH_2_), 34.1 (Cy CH_2_), 26.0 (Cy CH_2_), 25.8 (Cy CH_2_), 25.5 (Cy CH_2_); Anal. calcd. for C_20_H_29_ClN_2_Ni: C, 61.34; H, 7.46; N, 7.15; found: C, 61.10; H, 7.41; N, 6.84.

**[NiCl(Cp)(IDD)].** In the glovebox, a microwave vial with stir bar was charged with nickelocene (100.0 mg, 0.529 mmol, 1.5 equiv), IDD·HBF_4_ (172.6 mg, 0.353 mmol, 1 equiv) and [Et_4_N]Cl (58.4 mg, 0.352 mmol, 1 equiv) and the cap was secured with parafilm. Outside the glovebox, under a flow of argon, anhydrous THF (5 mL) was added and the vial was sealed with a crimp cap. The reaction was heated to 110 °C for 20 min in the microwave, during which time the solution changed the colour from green to red. The solvent was removed and the residue was taken up in hot toluene and filtered. The volume was reduced to ca. 1 mL, and then hexane was added to precipitate the product. The green solution was decanted, and the solid was washed with hexane and dried under high vacuum, to yield the product as a pink powder. Yield: 81.9 mg (0.146 mmol, 41%). ^1^H NMR (CDCl_3_, 400 MHz) δ_H_ 6.95 (s, 2H, N(CH)_2_N), 6.28 (app. quint., ^2^*J*_HH_ = 5.9 Hz, 2H, NCHR_2_), 5.21 (s, 5H, CpH), 2.09–1.91 (m, 4H, CH_2_), 1.91–1.73 (m, 6H, CH_2_), 1.73–1.39 (m, 34H, CH_2_); ^13^C{^1^H} NMR (CDCl_3_, 150 MHz) δ_C_ 158.0 (NCN), 119.6 (N(CH)_2_N), 91.8 (Cp CH), 59.2 (NCHR_2_), 31.4 (CDD CH_2_), 31.2 (CDD CH_2_), 24.3 (CDD CH_2_), 24.1 (CDD CH_2_), 23.9 (CDD CH_2_), 23.81 (CDD CH_2_), 23.78 (CDD CH_2_), 23.5 (CDD CH_2_), 22.5 (CDD CH_2_), 22.1 (CDD CH_2_); Anal. calcd. for C_32_H_53_ClN_2_Ni: C, 68.64; H, 9.54; N, 5.00; found: C, 68.54; H, 9.64; N, 4.88.

**General procedure for Suzuki–Miyaura reactions.** A reaction tube or Schlenk flask was charged with 4’-bromoacetophenone or 4’-chloroacetophenone (1 mmol), PhB(OH)_2_ (1.3 mmol, 1.3 equiv), nickel complex and K_3_PO_4_ (2.6 mmol, 2.6 equiv) and closed with a septum. Anhydrous toluene (3 mL) was added via syringe, and the vial was inserted into a pre-heated oil bath and stirred vigorously. The septum was removed and the reaction mixture was filtered through celite and stripped of solvent. A sample of this was then taken up in chloroform-*d* for ^1^H NMR spectroscopic analysis. Conversion was assessed from the relative integrals of the resonances corresponding to the product at δ_H_ = 2.63 ppm (4’-phenylacetophenone) and the starting material at δ_H_ = 2.56 ppm (4’-bromoacetophenone) or δ_H_ = 2.58 ppm (4’-chloroacetophenone). Slightly lower (by ca. 5%) conversions were obtained in Schlenk flasks versus sealed tubes.

## Supporting Information

File 1 Crystal structure data for new complexes.

File 2 NMR spectra for compounds and complexes.
