# Supplementary material for: Half-sandwich nickel(II) complexes bearing 1,3-di(cycloalkyl)imidazol-2-ylidene ligands
Source: Beilstein J Org Chem. 2015 Nov 12;11:2171–8. doi: 10.3762/bjoc.11.235 (PMC4660989; doi:10.3762/bjoc.11.235)

Supporting Information

for

Half-sandwich nickel(II) complexes bearing 1,3-di(cycloalkyl)imidazol-2-ylidene ligands

Johnathon Yau, Kaarel E. Hunt, Laura McDougall, Alan R. Kennedy and David J. Nelson*

Address: WestCHEM Department of Pure and Applied Chemistry, University of Strathclyde, Thomas Graham Building, 295 Cathedral Street, Glasgow G1 1XL, UK

Email: David J. Nelson - david.nelson@strath.ac.uk

* Corresponding author

NMR spectra for compounds and complexes

NMR Spectra

IDD·HBF$_4$ ^1H NMR (400 MHz, $CDCl_3$)

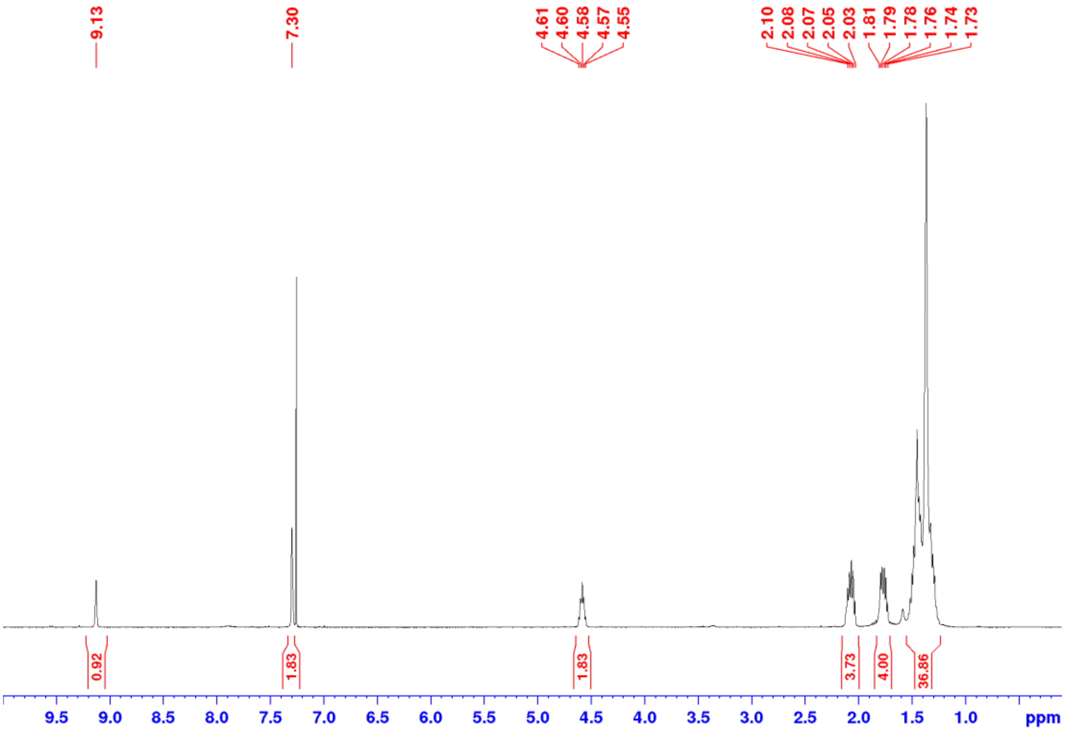

IDD·HBF$_4$ $^{13}C\{^1H\}$ NMR (151 MHz, $CDCl_3$)

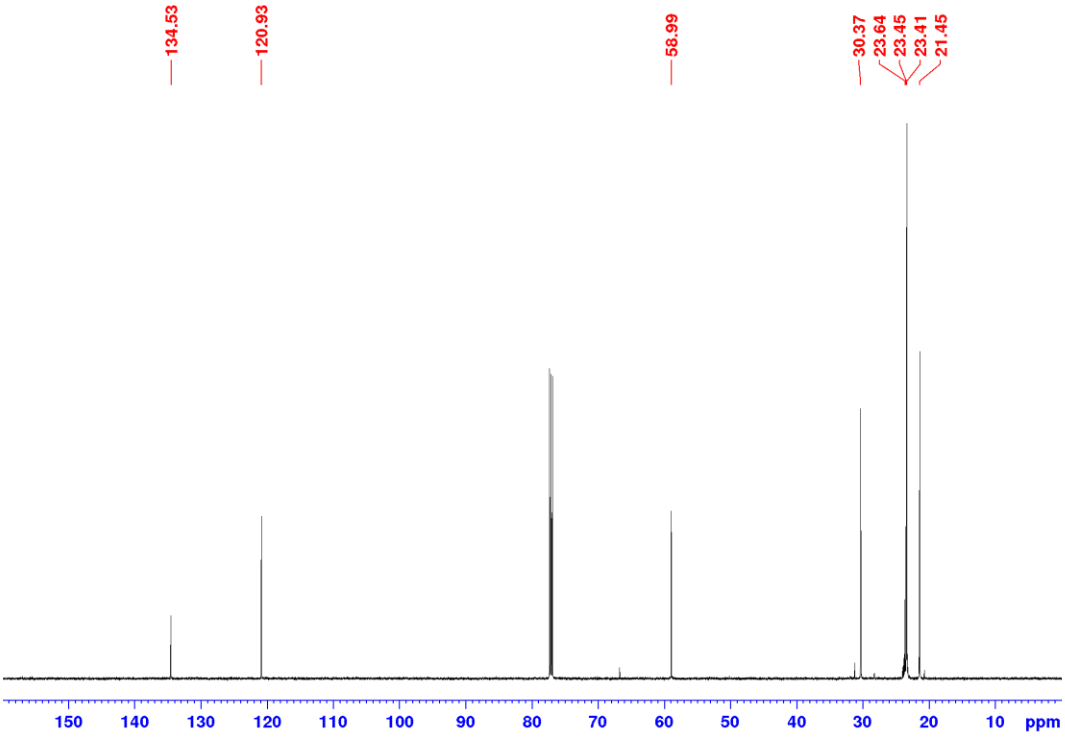

[NiCl(Cp)(ICy)] *^{1}H NMR (400 MHz, $CDCl_3$)*

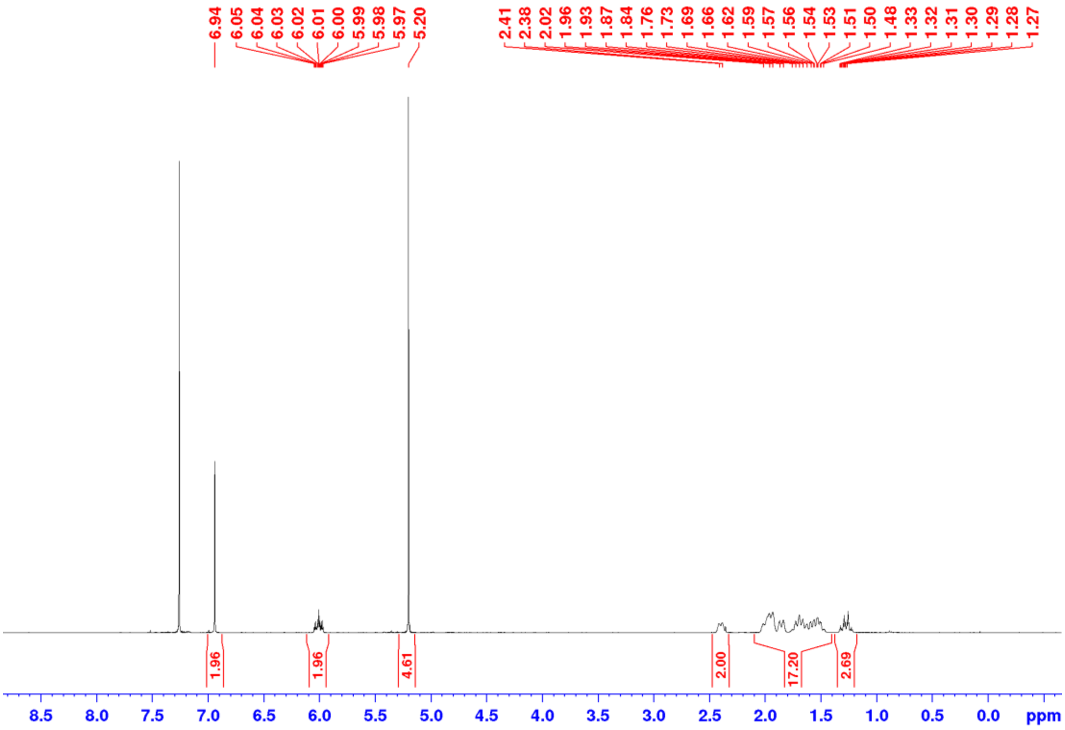

[NiCl(Cp)(ICy)] *$^{13}C\{^{1}H\}$ NMR (101 MHz, $CDCl_3$)*

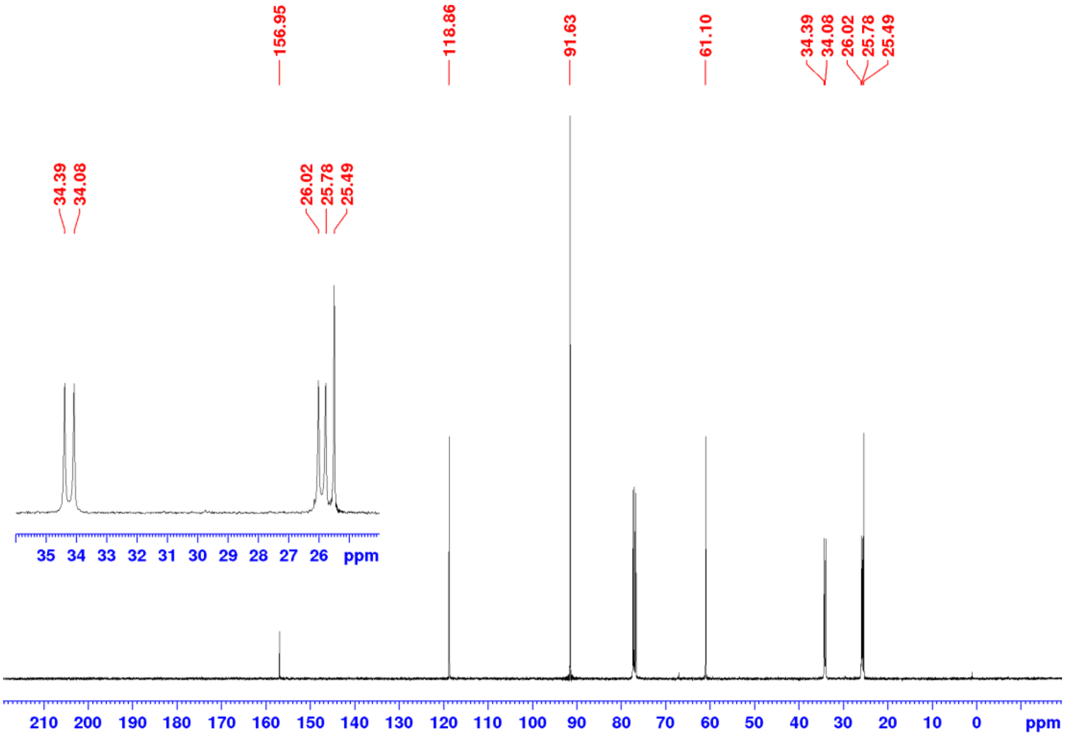

[NiCl(Cp)(IDD)] *^{1}H NMR (400 MHz, $CDCl_3$)*

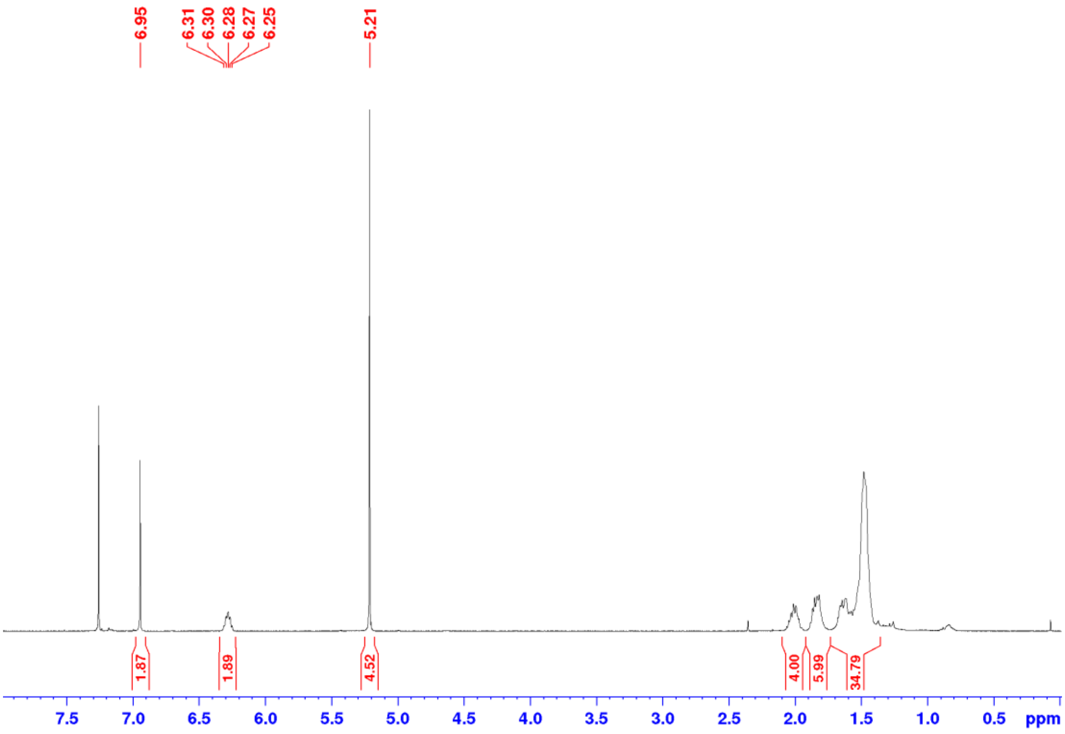

[NiCl(Cp)(IDD)] *^{13}C{^{1}H} NMR (151 MHz, $CDCl_3$)*

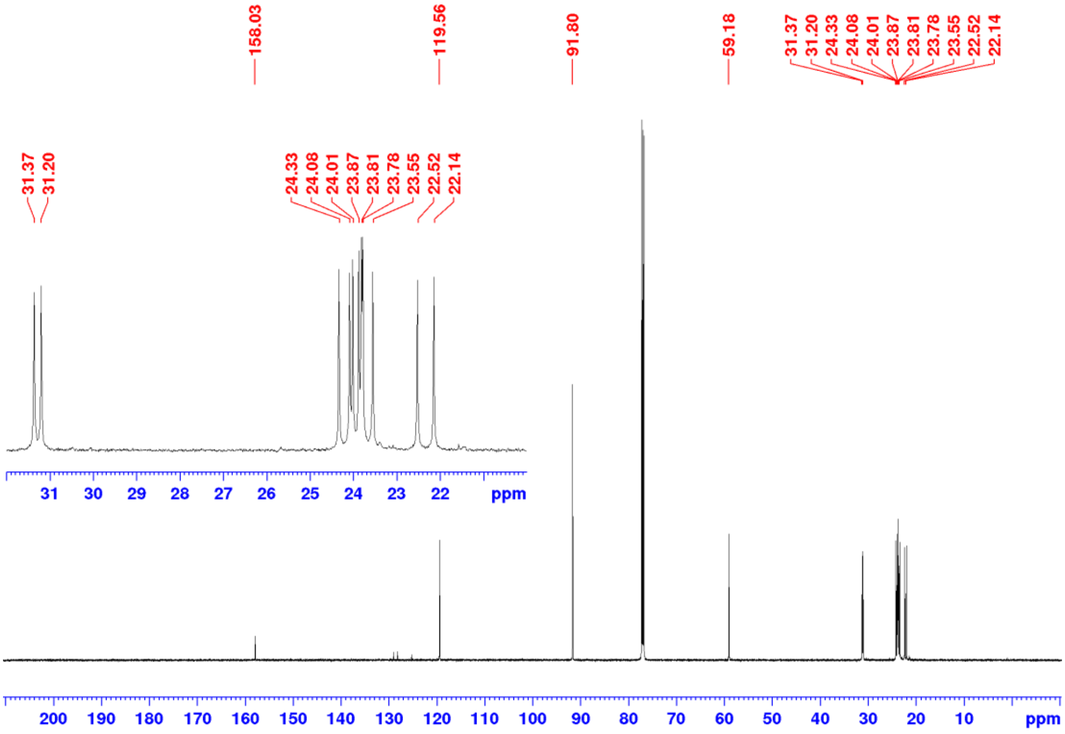

Supplement: File 2 — NMR spectra for compounds and complexes. [file Beilstein_J_Org_Chem-11-2171-s002.pdf]
